# p53-PHLDA3-Akt Network: The Key Regulators of Neuroendocrine Tumorigenesis

**DOI:** 10.3390/ijms21114098

**Published:** 2020-06-08

**Authors:** Yu Chen, Rieko Ohki

**Affiliations:** Laboratory of Fundamental Oncology, National Cancer Center Research Institute, Tsukiji 5-1-1, Chuo-ku, Tokyo 104-0045, Japan; ychen@ncc.go.jp

**Keywords:** p53, Akt, PHLDA3, neuroendocrine tumor

## Abstract

*p53* is a well-known tumor suppressor gene and one of the most extensively studied genes in cancer research. p53 functions largely as a transcription factor and can trigger a variety of antiproliferative programs via induction of its target genes. We identified *PHLDA3* as a p53 target gene and found that its protein product is a suppressor of pancreatic neuroendocrine tumors (PanNETs) and a repressor of Akt function. *PHLDA3* is frequently inactivated by loss of heterozygosity (LOH) and methylation in human PanNETs, and LOH at the *PHLDA3* gene locus correlates with PanNET progression and poor prognosis. In addition, in *PHLDA3*-deficient mice, pancreatic islet cells proliferate abnormally and acquire resistance to apoptosis. In this article, we briefly review the roles of p53 and Akt in human neuroendocrine tumors (NETs) and describe the relationship between the p53-PHLDA3 and Akt pathways. We also discuss the role of PHLDA3 as a tumor suppressor in various NETs and speculate on the possibility that loss of PHLDA3 function may be a useful prognostic marker for NET patients indicating particular drug therapies. These results suggest that targeting the downstream PHLDA3-Akt pathway might provide new therapies to treat NETs.

## 1. Introduction

*p53* is a well-known tumor suppressor gene that is mutated in more than half of human cancers of various types and contributes to their development [1,2,3,4]. p53 is a transcription factor that induces its target genes in accordance with the type and intensity of cell damage. Since the transactivation function of p53 is essential to its ability to suppress cancer, the identification and characterization of its target genes and associated pathways is critical to understanding p53-mediated tumorigenesis. We have previously identified several novel p53 target genes and downstream pathways that modulate tumorigenesis: *PHLDA3*, *PHLDA1*, *IER5*, *AEN*, *FUCA1*, *RPRM*, and *NOXA* [5,6,7,8,9,10,11,12,13,14]. In this review, we will focus on *PHLDA3*, a tumor suppressor gene that encodes a repressor of the Akt oncoprotein and is important for the development of neuroendocrine tumors (NETs). We will summarize the function of the p53-PHLDA3-Akt axis in NETs and discuss the potential importance of PHLDA3 in NET diagnosis and treatment.

## 2. p53-PHLDA3-Akt Axis

### 2.1. PHLDA3, a Target Gene of p53

*p53* is a well-known tumor suppressor gene normally present in all human cells that is usually activated by various types of stress, such as DNA damage, hypoxia, or oncogene activation [15,16,17,18,19,20,21,22]. In response to these signals, p53 undergoes various post-translational modifications including phosphorylation and acetylation and both its expression levels and subcellular localization are altered. Consequently, p53 binds to the promoters of its various target genes and activates their transcription [16,23,24,25,26]. The effector functions of p53 have yet to be fully elucidated, and a comprehensive identification and functional analysis of p53 target genes remains a critical research goal.

In previous studies we comprehensively screened genes that are directly induced by p53 and focused in particular on genes of hitherto unknown function. Among these genes, we were particularly interested in *PHLDA3*, which is strongly induced by wild-type p53 [13,27,28]. In-silico analysis revealed that there is a p53 binding site near the *PHLDA3* gene transcription site, and ChIP-chip analysis and ChIP-sequence analysis confirmed that p53 binds to this sequence [13]. These results thus identified *PHLDA3* as a p53 target gene.

### 2.2. Oncogene Akt and p53-Akt Network

*Akt* is a particularly well-known oncogene that is abnormally activated in many tumors by a variety of extracellular stimuli, such as growth factors and hormones [29,30,31,32]. Akt activation occurs downstream of phosphoinositide 3-kinase (PI3K), a lipid kinase that catalyzes the phosphorylation of phosphatidylinositol 4,5-bisphosphate (PIP_2_) to produce phosphatidylinositol 3,4,5-trisphosphate (PIP_3_) and is linked to cellular transformation and human cancers [33,34]. The activation of Akt requires the binding of PIP_3_ and this binding is mediated by a Pleckstrin Homology (PH) domain found in the amino terminus of Akt [35]. When PIP_3_ appears in the membrane, Akt can re-localize to the membrane by binding to PIP_3_ via its PH domain, and this induces Akt phosphorylation and consequent activation. Activated Akt is a serine threonine kinase that regulates cell proliferation by phosphorylating various downstream substrates such as Mouse Double Minute 2 homolog (MDM2), Glycogen Synthase Kinase 3 (GSK3), Tuberous Sclerosis Complex 2 (TSC2), and Mechanistic Target of Rapamycin Complex 1 (mTORC1), and this regulation plays a central role in signaling required for cell survival [33,36].

Since p53 and Akt are major but opposing regulators in the signaling pathways determining cell survival and death, it is not surprising that there is a signaling network integrating the functions of these two proteins [37,38,39,40]. For example, activated Akt negatively regulates p53 expression by phosphorylating MDM2, which translocates to the nucleus and facilitates p53 degradation [41]. On the other hand, p53 induces the expression of PTEN, which terminates PI3K/PIP_3_ signaling by dephosphorylating PIP_3_ and thereby inhibiting Akt activation. Disruption of the p53-Akt network resulting from the functional loss of p53 or PTEN or the over-activation of Akt or MDM2 has been frequently observed in cancer cells [2,29,30,42,43,44,45]. This suggests that the balance of the p53-Akt network plays an essential role in tumorigenesis and could be one of the most important targets in cancer therapy.

### 2.3. PHLDA3 Functions as an Endogenous Dominant Negative Regulator of Akt

**Figure 1:**
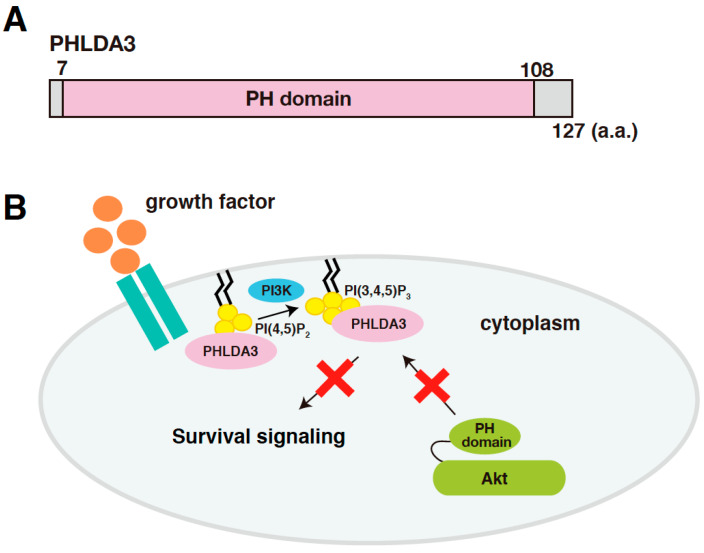
*PHLDA3* is a target gene of p53 and encodes a protein that functions as an endogenous inhibitor of Akt. (**A**) A schematic diagram of PHLDA3. Pleckstrin Homology (PH) domain is the only known protein domain in PHLDA3. (**B**) A model of Akt repression by PHLDA3. PHLDA3 functions as a dominant negative repressor of Akt. PHLDA3 localizes to the plasma membrane via binding to phosphatidylinositol phosphates (PIPs), thereby competing with Akt binding to PIPs and suppressing Akt-mediated survival signaling.

*PHLDA3* (Pleckstrin Homology Like Domain Family A Member 3) is a gene that encodes a protein consisting of 127 amino acids, most of which consists of a PH domain (amino acids 7-108; Figure 1A). PHLDA3 binds to various phosphatidylinositol phosphates (PIPs) through this domain and thereby localizes to the cell membrane. Overexpression of PHLDA3 results in activation of caspase-3 and increased cell death, indicating that PHLDA3 can induce apoptosis. Akt also possess a PH domain, and we found that PHLDA3 suppresses Akt activation by competitively inhibiting the binding of Akt to PIPs. Thus, PHLDA3 functions as an endogenous dominant-negative repressor of Akt that blocks Akt-mediated survival signaling by suppressing Akt activation (Figure 1B). The identification and analysis of PHLDA3 has revealed a novel p53 downstream pathway that suppresses Akt’s oncogenic function [13].

## 3. *p53* Mutations in Neuroendocrine Tumors

### 3.1. Neuroendocrine Tumors

Neuroendocrine tumors (NETs) are a relatively rare and heterogeneous tumor type [46,47,48,49]. Neuroendocrine cells are distributed throughout the entire body, and NETs have been found in lung, pancreatic islet, pituitary, thyroid gland, parathyroid gland, rectum, and many other neuroendocrine tissues. NETs are generally low-grade tumors with a good prognosis and low risk of distant metastases. Histopathological classification is important in determining the nature of the NET, its prognosis, and recommended therapies. NETs synthesize and secrete peptide hormones and depending on the effects of these tumor-secreted hormones on the body, NETs are classified into functional NETs with abnormal hormonal symptoms and non-functional NETs without any symptoms. Functional NETs cause a variety of symptoms depending on the type of hormone being over-produced. Non-functional NETs usually are a larger size and often compress nearby organs. 

There is a relatively higher incidence of NETs derived from lung and pancreatic islet compared to other sites [48]. Nearly one-fourth of lung neoplasms are lung NETs. Based on the cell shape, Lung NETs are divided into two types, carcinoids and neuroendocrine carcinomas. In addition, carcinoids are categorized into low-grade typical carcinoids (TC) and atypical carcinoids (AC), and neuroendocrine carcinomas are divided into high-grade small-cell lung cancer (SCLC) and large-cell neuroendocrine carcinomas (LCNEC).

Pancreatic neuroendocrine tumors (PanNETs) are the second most common type of pancreatic epithelial tumor, with an incidence of 2~3 per 100,000 [50]. PanNETs tend to grow slowly and can possibly spread to other parts of the body. The 2017 WHO classification grouped well-differentiated PanNETs into various grades depending on their Ki-67 proliferation index, i.e., Grade 1 (Ki-67 index <3%), Grade 2 (Ki-67 index 3~20%), and Grade 3 (Ki-67 index >20%). Poorly-differentiated pancreatic neuroendocrine carcinomas (PanNECs) usually have a Ki-67 proliferation index of more than 20%, tend to grow and spread quickly, and can spread to other parts of the body. It has been reported that about 85% of PanNETs are nonfunctional tumors, about 10% are insulinomas, which are the most common type of functional PanNET and produce insulin, and the remaining PanNETs produce other hormones such as glucagon, somatostatin, or gastrin [51,52].

The gastrointestinal (GI) tract is another site where most of the NETs develop in. Rectal NETs are the most common type of GI-NET, with a 5-year overall survival rate of 88% [53,54,55]. Rectal NETs are classified into grade 1 (Ki-67 index<3%), grade 2 (Ki-67 index 3~20%), and grade 3 (Ki-67 index >20%) according to the 2016 WHO classification. 

Although the incidence of NETs has strikingly increased in the last decades, there are still limited data about the pathogenesis and treatment in NETs. The mechanisms elucidation of NET tumorigenesis and the development of a prognostic marker may be helpful in predicting tumor behavior and guiding therapy.

### 3.2. p53 Mutations Are Rare in Lung Neuroendocrine Tumors (NETs), Pancreatic Neuroendocrine Tumors (PanNETs), and Rectal NETs 

As NETs are rare cancers and very difficult to study, the mechanisms of NET tumorigenesis and development are still not well understood. It has been reported that *p53* mutations occur in many types of cancer and are associated with poorer clinical outcomes, greater resistance to treatment, and higher degrees of metastases [2,56,57,58]. Several groups have investigated the status of p53 in NET specimens and shown that *p53* mutations are infrequent in low-grade lung NETs [59,60,61]. More recently, Vollbrecht et al. looked for mutations in 221 mutational hot spots within 48 tumor-relevant genes in lung NETs and found that *KIT*, *PTEN*, *HNF1A*, and *SMO* were altered in ACs, while the *SMAD4* mutation was found in TC subtypes. However, they did not find any *p53* mutations in these specimens [62]. In addition, Simbolo et al. screened 148 lung NETs, consisting of 53 TCs, 35 ACs, 33 SCLCs, and 27 LCNECs, using next-generation sequencing and whole-exome sequencing and found *MEN1* alterations are almost exclusive to low-grade carcinoids, whereas alterations in both *p53* and PI3K/Akt/mTOR pathway genes were found more commonly in carcinomas, which are a higher grade of NET [63]. In 2018, George et al. performed a comprehensive genomic and transcriptomic analysis of 75 LCNECs and identified two molecular subgroups: “type I LCNECs” and “type II LCNECs”, both of which are enriched for inactivated *p53* [64]. In summary, *p53* mutations are frequently found in high-grade carcinomas, but are rarely found in low-grade carcinoids.

Mutations of *p53* are also rare in PanNETs [65,66]. Jiao et al. screened the most commonly mutated cancer genes, including *p53*, in well-differentiated somatic PanNETs by whole exome sequencing and found a high frequency of mutations in *MEN1* (multiple Endocrine Neoplasia Type 1), *ATRX* (alpha thalassemia/mental retardation syndrome X-linked), and *DAXX* (death-domain associated protein), but a low frequency of mutations in *p53* [67]. Recently, Scarpa et al. have performed whole-genome sequencing and found that clinically sporadic PanNETs contain frequent germline mutations, including mutations in *MUTYH*, *CHEK2*, and *BRCA2*, which are the DNA repair genes. They reported that sporadic PanNETs also usually bear mutations in *MEN1*, the chromatin remodeling pathway (*SETD2*, *MLL3*), the telomere maintenance pathway (*DAXX*, *ATRX*) and activators of the mTOR signaling pathway (*PTEN*, *DEPDC5*, *TSC1*, *TSC2*), but rarely have mutations in *p53*. In addition to these gene mutations, they also identified a subgroup of tumors that are associated with hypoxia and HIF signaling [50].

There is also limited data about the incidence of driver mutations in rectal NETs. Ha et al. sequenced 69 primary low-grade rectal NETs and found only 10% of these tumors bore *p53* mutations [68].

Therefore, while mutations of *p53* are frequently found in many cancer types, they are rarely observed in low-grade lung NETs, PanNETs, and rectal NETs.

## 4. PHLDA3 Functions as a Tumor Suppressor in Various NETs

### 4.1. Functional Loss of PHLDA3 Is Frequently Found in Lung NETs

Abnormal activation of the PI3K/Akt pathway is observed in many cases of lung cancer. Since PHLDA3 is a repressor of Akt, it is conceivable that functional loss of PHLDA3 could contribute to the tumorigenesis and development of lung cancers. We analyzed various types of lung cancers and found a high frequency of *PHLDA3* gene defects in lung NETs compared to other types of lung cancer [13]. In these lung NETs, *PHLDA3* gene expression was lower and Akt activation was higher compared to normal tissues, raising the possibility that loss of PHLDA3 function caused Akt activation in these cancers.

The PTEN tumor suppressor is an upstream factor and major suppressor of the Akt pathway. It has been shown that PHLDA3 and PTEN inhibit the PI3K/Akt pathway by different mechanisms. We also found that in LCNEC samples, loss of *PTEN* and loss of *PHLDA3* are not mutually exclusive, but rather are additive with respect to Akt activation [13].

**Figure 2:**
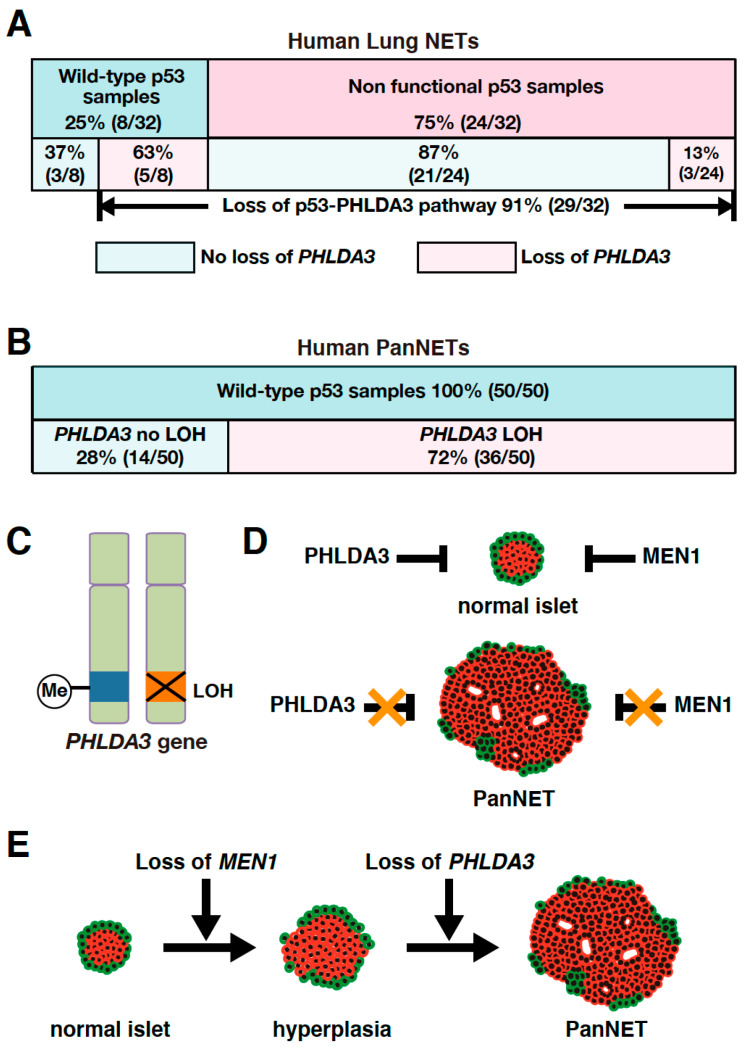
*PHLDA3* gene defects are observed in lung and pancreatic neuroendocrine tumors (NETs). (**A**) Among lung NETs having wild-type p53, 63% exhibited loss of *PHLDA3*, whereas among lung NETs having a nonfunctional p53, only 13% exhibited loss of *PHLDA3*. Nonfunctional p53 can be caused by deletion as well as mutation. Abnormalities in *p53* can result in upregulated expression of the protein, which may be detected by immunohistochemistry. Genomic sequencing is also preformed to analyze *p53* mutations. Chromosome copy number alterations in *PHLDA3* are analyzed by comparative genomic hybridization (CGH). (**B**) High frequency of *PHLDA3* loss of heterozygosity (LOH) is found in pancreatic neuroendocrine tumors (PanNETs), which commonly have wild-type p53. (**C**) Two-hit inactivation of *PHLDA3* in PanNETs. One of the *PHLDA3* loci undergoes LOH and the other undergoes methylation. (**D**) PanNET tumorigenesis requires the functional loss of both PHLDA3 and MEN1. PHLDA3 and MEN1 suppress cell proliferation in normal islet cells. Loss of both PHLDA3 and MEN1 function is necessary for PanNET tumorigenesis. (**E**) Functional loss of PHLDA3 contributes to PanNET progression. Loss of MEN1 function leads to islet hyperplasia and/or atypia over time, and additional loss of PHLDA3 function is required for tumor formation and progression.

As *PHLDA3* is a p53 target gene, we analyzed the association between *PHLDA3* loss and p53 status in these lung NET specimens. We found that among the lung NETs having wild-type p53, 63% exhibited *PHLDA3* loss, whereas among specimens having a nonfunctional p53 only 13% exhibited *PHLDA3* loss (Figure 2A). Collectively, 91% of lung NETs have a functional loss of either p53 or PHLDA3 (Figure 2A), suggesting that defects in this p53-PHLDA3 pathway play a major role in lung NET tumorigenesis [13].

### 4.2. PHLDA3 Is a Tumor Suppressor of PanNETs

#### 4.2.1. *PHLDA3* Is Inactivated by Both Loss of Heterozygosity (LOH) and Methylation in Human PanNETs

It has been reported that loss of heterozygosity (LOH) is frequently found at the 1q31 locus in PanNETs [69,70]. Since the *PHLDA3* gene is located at 1q31 and LOH at the *PHLDA3* gene locus is frequently found in lung NETs, we speculated that the *PHLDA3* gene may also undergo LOH in PanNETs. We therefore analyzed the LOH of the *PHLDA3* gene in PanNETs using a microsatellite marker near the *PHLDA3* gene locus. We found that LOH at the *PHLDA3* gene locus was detected in 72% of human PanNETs (Figure 2B). We further found that *PHLDA3* gene expression level in PanNET specimens was significantly decreased in specimens that have LOH at the *PHLDA3* gene locus compared to those that do not. Since LOH involves the loss of one allele, we analyzed the status of the remaining allele in PanNET specimens and found that the remaining *PHLDA3* allele invariably underwent methylation in its transcriptional regulatory region. Thus, PanNETs are characterized by a two-hit inactivation of the *PHLDA3* gene, i.e., LOH and methylation (Figure 2C). These results identify *PHLDA3* as a novel tumor suppressor gene of PanNETs [14].

#### 4.2.2. Functional Loss of Both PHLDA3 and MEN1 Is Essential for PanNET Tumorigenesis

The LOH frequency of the *PHLDA3* gene is similar to that reported for the *MEN1* gene, another important tumor suppressor gene in PanNETs. The *MEN1* gene is known to be causative for multiple endocrine neoplasia type 1, which undergoes frequent LOH and mutation in PanNETs [50,67,71,72]. We therefore investigated the relationship between the *PHLDA3* gene and *MEN1* gene in PanNETs. We first performed LOH analysis of the *MEN1* gene and found that its frequency of LOH was 67% in human PanNETs. Interestingly, LOH at the *PHLDA3* and *MEN1* loci were not mutually exclusive, as would be expected if PHLDA3 and MEN1 were on the same tumor-suppressing pathway. We also observed a significantly high frequency of LOH at both the *PHLDA3* and *MEN1* loci. These results suggest that the functional loss of both the *PHLDA3* and *MEN1* genes is necessary for the development of human PanNETs and inactivation of these pathways cooperatively contribute to PanNET development (Figure 2D) [14].

#### 4.2.3. LOH at *PHLDA3* Locus Is Related to PanNET Malignancy and Prognosis

The *PHLDA3* gene locus has been reported to have a high frequency of LOH in PanNETs and correlate with tumor progression [14,69,70]. We next examined the relationship between LOH at the *PHLDA3* gene locus, tumor malignancy, and patient prognosis. We observed that PanNETs with LOH at the *PHLDA3* gene locus displayed a higher malignancy, and prognosis was worse versus those without LOH. On the other hand, LOH at the *MEN1* gene locus was not associated with tumor malignancy or prognosis. These results suggest that the *PHLDA3* gene pathway suppresses PanNET progression (Figure 2E) [14].

### 4.3. The Function of PHLDA3 in Other NETs

In addition to human lung NETs and PanNETs, the incidence of rectal NETs has been increasing in recent years [55,73,74,75]. In many cases, rectal NETs are very small lesions and very difficult to find unless accompanied by lymph node metastasis, and further research is needed to clarify the risk factors for rectal NET tumorigenesis and lymph node metastasis. We analyzed PHLDA3 status in 55 rectal NET specimens and reported that 60% of them showed LOH at the *PHLDA3* gene locus. This indicates that PHLDA3 has an important function in tumor suppression in rectal NETs. In addition to *PHLDA3*, a high frequency of LOH was also observed at the *MEN1* gene locus in these specimens. LOH at the *PHLDA3* and *MEN1* loci were not mutually exclusive and occurred together in both loci at a high frequency. Thus, similar to PanNETs, the tumor suppressing pathways involving PHLDA3 and MEN1 are distinct in rectal NETs, and their development involves the functional loss of both pathways [11].

We therefore regard PHLDA3 as a common tumor suppressor of various NETs, although analysis of NETs from other neuroendocrine tissues is still required to be conclusive.

## 5. Functional Analysis of PHLDA3 Using a *PHLDA3*-Deficient Mouse Model

### 5.1. Functional Loss of PHLDA3 Induces Akt Pathway Activation and Cell Proliferation in Islet β Cells

PanNETs are tumors that develop from pancreatic islet cells. Previous studies have shown the proliferation of pancreatic islet β cells involves the Akt pathway activated by glucose and/or growth factor stimulation [76]. Moreover, Bernal-Mizrachi et al. have noted that transgenic mice expressing a constitutively activated Akt in pancreatic islet β cells exhibit an increase in islet mass due to enhanced proliferation of β cells [77]. These results suggest that the Akt pathway plays a central role in promoting cell proliferation and inhibiting cell death in islet β cells. Since PHLDA3 inhibits Akt activation, we analyzed PHLDA3 function in islet cells using normal rat islet cells and RIN cells derived from a rat insulinoma. Inhibition of *PHLDA3* gene expression enhanced Akt activation and promoted cell proliferation in these cells. In addition, overexpression of the *PHLDA3* gene in MIN6 cells, a cell line derived from mouse pancreatic β cells, led to decreased Akt activity and phosphorylation of factors downstream of Akt. These results show that the *PHLDA3* gene negatively regulates Akt pathway activation and cell proliferation in islet β cells [14].

### 5.2. Functional Loss of PHLDA3 Induces Hyperplasia of Pancreatic Islets and Increased Insulin Secreting

**Figure 3:**
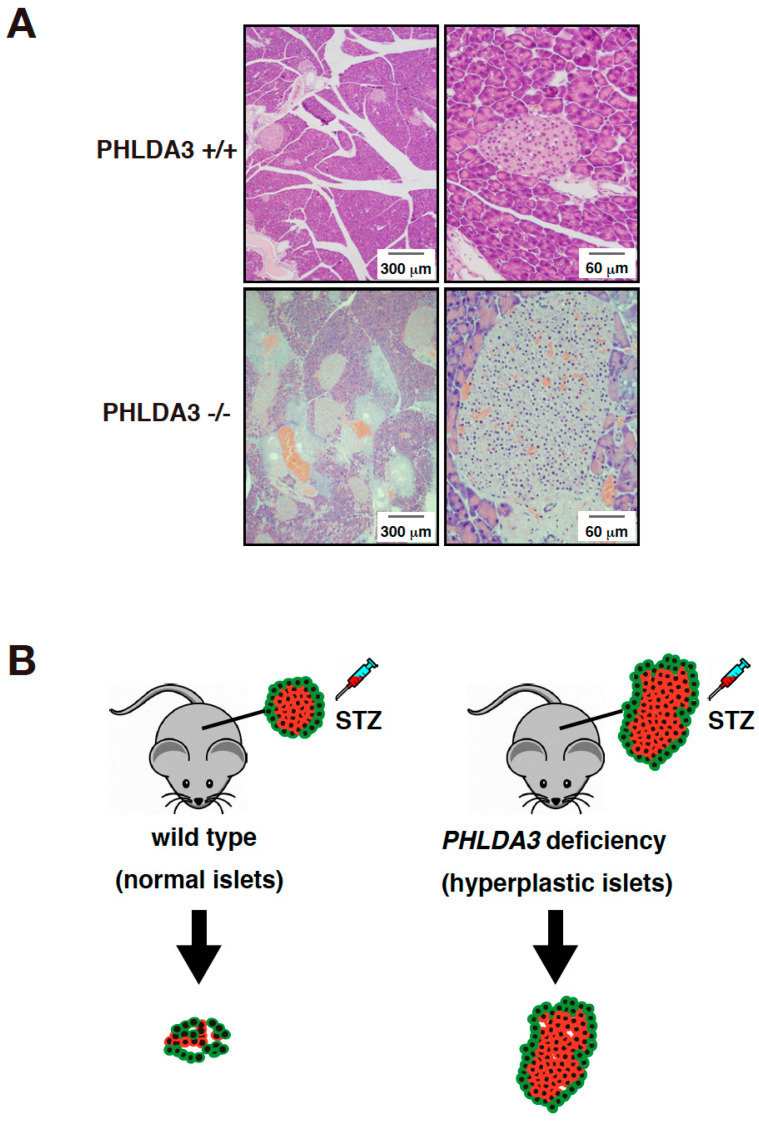
*PHLDA3*-deficient mice exhibit islet hyperplasia and resistance of β cells to apoptosis. (**A**) HE staining of islets from wild type and *PHLDA3*-deficient 10 month-old mice. (**B**) Apoptosis of β cells is observed in wild type mice treated with streptozotocin (STZ) but is suppressed in *PHLDA3*-deficient mice.

In order to analyze the function of PHLDA3 in vivo, islet cells from *PHLDA3*-deficient mice were analyzed for activation of Akt and its downstream substrates. We found that phosphorylation of Akt and its downstream substrates such as p70S6 kinase, ribosomal protein S6, GSK-3β, and MDM2 increased, indicating that the Akt pathway is abnormally activated by PHLDA3 deficiency. Since the activation of Akt pathway is known to cause proliferation of islet β cells and hyperplasia of pancreatic islets [77,78], we examined the pancreas of wild-type and *PHLDA3*-deficient mice and found that pancreatic islets taken from 10-month-old *PHLDA3*-deficient mice displayed islet hyperplasia (Figure 3A).

Pancreatic islets contain several types of cells, such as β cells that produce insulin and α cells that produce glucagon. When pancreatic sections from wild-type and *PHLDA3*-deficient mice were double-stained for insulin and Ki-67, a cell proliferation marker, it was found that most Ki-67-positive cells were β cells in *PHLDA3*-deficient islets, indicating that β cell proliferation is enhanced in *PHLDA3*-deficient islets. Since β cells produce and secrete insulin, which regulates blood glucose levels, we measured the plasma insulin levels and the blood glucose levels in wild-type and *PHLDA3*-deficient mice. We found higher plasma insulin and lower blood glucose levels in *PHLDA3*-deficient mice compared with wild-type mice. These results show that loss of *PHLDA3* expression results in enhanced proliferation of islet β cells and increased insulin secretion.

As it is also known that cells become enlarged when Akt is activated, we checked the size of the islet cells from *PHLDA3*-deficient mice and found that β cells are indeed larger than those from wild-type mice. These data show that functional loss of PHLDA3 leads to β cell hypertrophy.

Taken together, these results show that loss of *PHLDA3* induces Akt hyperactivation leading to the enhanced proliferation and enlargement of β cells, increased insulin production, and pancreatic islets hyperplasia [14].

### 5.3. PHLDA3 Deficiency Causes Apoptosis Resistance in Pancreatic Islet β Cells

Streptozotocin is a drug that induces apoptosis specifically in pancreatic islet β cells and is used experimentally to cause type 1 diabetes in animal models [79]. We found that when streptozotocin was administered to wild-type and *PHLDA3*-deficient mice, the resulting increase in blood glucose levels was significantly suppressed in *PHLDA3*-deficient mice compared to wild-type mice. In addition, the decrease in β cells was dramatically suppressed (Figure 3B). These results show that PHLDA3 deficiency causes islet β cells to become resistant to apoptosis [14].

### 5.4. PHLDA3-Deficient Islets May Have Application in Diabetes Therapy

Interestingly, *PHLDA3*-deficient mice do not show increased tumor development, even though PHLDA3 deficiency activates the PI3K/Akt/mTOR pathway and promotes the proliferation of islet β cells, increased insulin production and decreased apoptosis. Another characteristic of PHLDA3 deficiency is increased resistance to stress such as hypoxia, which is a problem in islet transplantation, compromising islet isolation and inducing damage during the early stages of transplantation [10]. We have found that transplantation of *PHLDA3*-deficient islets into a diabetic mouse model leads to a better outcome than those transplanted with wild-type islets [10]. These results suggest that PHLDA3 deficiency contributes to islet cell survival, suggesting that such cells could be useful in the treatment of diabetes by islets transplantation. We are hopeful that the transplantation of *PHLDA3*-deficient islets may be used as a novel therapy for the treatment of diabetes in the future.

## 6. Conclusions

We have shown that the *PHLDA3* gene is a p53 target gene and encodes a repressor of Akt. In addition to our works, several other groups have described the significance of *PHLDA3* as a p53 target gene. For example, Allen et al. have revealed that *PHLDA3* is one of the genes most strongly induced by p53 [27]. Brady et al. have found that *PHLDA3* is one of the most important p53 target genes involved in tumor suppression [28]. In our studies, we have shown that PHLDA3 is a tumor suppressor of human PanNETs, and loss of PHLDA3 function is achieved by 2-hit inactivation in PanNETs, i.e., LOH and methylation. PHLDA3 has been found to be functionally deficient not only in PanNETs but also in lung NETs and rectal NETs, suggesting that it may be a common tumor suppressor of NETs [11,13,14]. Analysis of the function of PHLDA3 in other types of NETs including pituitary NETs, esophageal NETs, thyroid NETs, and parathyroid NETs will be important in future studies. Although we expect this area of research will be of interest to numerous researchers, most of the results to date come from our own studies. Further studies by many researchers, including our group, will be necessary to assess the functions of PHLDA3 in tumorigenesis.

Although *PHLDA3* is a target gene regulated by p53, the mutation of *p53* itself is rarely found in several NETs including low-grade lung NETs, PanNETs, and rectal NETs. We have noted that functional loss of PHLDA3 and p53 occur in a mutually exclusive pattern in lung NETs and PanNETs (Figs. 2A, 2B). This indicates that PHLDA3 is an important downstream tumor-suppressive mediator of p53, and its functional loss is critical to tumorigenesis in NETs having wild-type p53. Further analysis should clarify whether loss of p53 and PHLDA3 function also occur in a mutually exclusive pattern in other types of NETs, which would confirm the importance of the p53-PHLDA3 pathway in NET tumorigenesis. We would also note that there are many other types of cancer besides NETs that retain wild-type p53 [80,81]. Therefore, it would be of great interest to ask if PHLDA3 function is defective in these cancers. We could imagine that PHLDA3 plays a role as a tumor suppressor in a broad range of cancers having wild-type p53, and this p53-PHLDA3 tumor-suppressive pathway could be a novel target for cancer therapy.

We also have showed that functional loss of PHLDA3 in PanNETs correlates with higher malignant tumor progression and poorer patient prognosis. These results indicate that PHLDA3 is very important in the progression of PanNETs and suggest that analysis of *PHLDA3* LOH in PanNETs could be a useful diagnostic marker providing information regarding the tumor’s metastatic potential and patient prognosis.

In recent years, streptozotocin has been used as a PanNET therapy [82,83,84,85,86]. However, islet β cells from *PHLDA3*-deficient mice were found to be resistant to streptozotocin [14]. Based on this result, PanNET patients having *PHLDA3* LOH may be expected to be non-responsive to streptozotocin therapy. Another effective drug for PanNETs is everolimus, a PI3K/Akt/mTOR pathway inhibitor [87]. We may expect everolimus to be more effective in PanNET patients having *PHLDA3* LOH, since loss of *PHLDA3* results in the abnormal activation of the PI3K/Akt/mTOR pathway. On the other hand, the PI3K/Akt/mTOR pathway has been reported to be activated in many other cancers [29,30,31]. Since PHLDA3 regulates the PI3K/Akt/mTOR pathway by suppressing Akt activation, it is possible that new drugs that mimic PHLDA3 function may show efficacy not only in PanNETs but also in other cancers in which Akt is highly activated.

**Figure 4:**
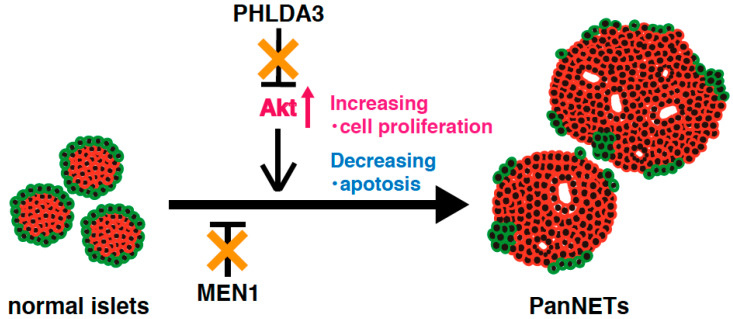
A model of NET tumorigenesis resulting from functional loss of both PHLDA3 and MEN1. PHLDA3 is a repressor of Akt. Loss of *PHLDA3* leads to Akt activation, increased cell proliferation and decreased apoptosis. PHLDA3 functions independent of MEN1, and PanNET tumorigenesis therefore requires the functional loss of both PHLDA3 and MEN1.

Analysis of a *PHLDA3*-deficient mouse model has revealed that Akt is abnormally activated due to functional loss of PHLDA3 in pancreatic islet β cells, leading to islet hyperplasia, but not to the development of PanNETs [14]. These results suggest that activation of Akt pathway alone is not sufficient to cause PanNETs. On the other hand, *MEN1* knockout mice develop low-grade PanNETs at a late stage. This delay in PanNET formation implies that additional abnormal factors and/or pathways are required for PanNET tumorigenesis [88]. In human PanNETs, deficiencies of both the PHLDA3 and MEN1 pathway are required for tumorigenesis and progression, indicating that the combined loss of PHLDA3 and MEN1 function promotes PanNET tumorigenesis (Figure 4). The above-mentioned results suggest that mice lacking both the *PHLDA3* and *MEN1* genes might be a good model for development of PanNETs at an early age, and for analyzing the mechanisms of PanNET tumorigenesis and progression.

In summary, *PHLDA3* is a p53 target gene that functions as an endogenic inhibitor of Akt. Functional loss of PHLDA3 is found in PanNETs, and LOH at *PHLDA3* locus is frequently found in lung and rectal NETs. Together these results suggest that loss of PHLDA3 function disrupts the balance of p53-PHLDA3-Akt axis and promotes NET tumorigenesis and progression.
